# A Novel Nomogram Predicting Distant Metastasis in T1 and T2 Gallbladder Cancer: A SEER-based Study

**DOI:** 10.7150/ijms.47073

**Published:** 2020-07-02

**Authors:** Yu-Long Cai, Yi-Xin Lin, Li-Sheng Jiang, Hui Ye, Fu-Yu Li, Nan-Sheng Cheng

**Affiliations:** Department of Biliary Surgery, West China Hospital, Sichuan University, Chengdu 610041, Sichuan Province, China.

**Keywords:** Nomogram, gallbladder cancer, SEER.

## Abstract

**Background:** Gallbladder cancer (GBC) is the most common malignancy of the biliary system. Early T stage GBC patients with distant metastasis are proven to have a worse prognosis. In this study, our aim was to construct and validate a novel nomogram for predicting distant metastasis in T1 and T2 GBC.

**Methods:** Between 2004 and 2014, patients with T1 and T2 GBC were identified in the Surveillance, Epidemiology, and End Results (SEER) database. All of the eligible patients were randomly divided into training and validation cohorts. Univariate and multivariate analyses were used to assess significant predictive factors associated with distant metastasis. A nomogram was developed and validated by a calibration curve and receptor operating characteristic curve (ROC) analysis.

**Results:** According to the inclusion and exclusion criteria, 3013 patients with historically confirmed AJCC stage T1 and T2 GBC were enrolled. Younger age, high pathological grade, nonadenocarcinoma, T1, N1 and larger tumor size correlated positively with the risk of distant metastasis. A novel nomogram was established to predict distant metastasis in early T stage GBC patients. Internal validation with a calibration plot in the training cohort showed that this nomogram was well calibrated. Through ROC curve analysis, the areas under the ROC curves in the training and validation cohorts were 0.723 and 0.679, respectively.

**Conclusions:** Although some limitations exist in this predictive model, the nomogram revealed the relationship between the clinicopathological characteristics of T1 and T2 GBC patients and the risk of distant metastasis. The novel nomogram will assist in patient counseling and guide treatment decision making for T1 and T2 GBC patients.

## Introduction

Gallbladder cancer (GBC) is the most common malignancy of the biliary system, representing 80-95% of all biliary tract cancers worldwide 1. Despite the development of diagnosis, surgery, and chemotherapy during the last decade, GBC remains an aggressive cancer with an overall dismal outcome 2. One reason for the unsatisfactory prognosis is the lack of effective screening and early symptoms, which contribute to the relatively delayed presentation of the disease. Nonetheless, GBC itself is a highly malignant tumor; thus, early distant metastasis usually occurs. For patients with resected T2 lesions, 16% have been found to have distant metastasis 3. Distant metastasis is most often found in the liver, lung, and peritoneum; the liver, an adjacent organ, is the leading metastatic site, accounting for over 50% of GBC patients with metastasis 4.

According to the American Joint Committee on Cancer (AJCC) staging system, distant metastasis (M1) with any T or any N stage is defined as Stage IV 5. The 5-year survival rate of Stage IV patients is only 1% 6. This trend suggests that distant metastasis often has a worse prognosis. Particularly for early T stage patients, such as T1 and T2, oncologists or hepatobiliary surgeons may overlook the possibility of distant metastasis, resulting in insufficient preoperative diagnostic examinations, follow-up, and postoperative comprehensive treatment. However, few studies to date have evaluated the risk factors or a predictive model of distant metastasis in GBC. A recent 10-institution study from America showed that advanced T-stage, grade, and presence of lymphovascular and perineural invasion were all associated with increased rates of distant metastasis 7. It is worth noting that substantial heterogeneity exists among GBC patients in terms of demographic and clinicopathological information, such as age, sex, pathological type, and tumor size. Therefore, a well-designed predictive model of distant metastasis in T1 and T2 GBC patients that covers all factors is needed.

A nomogram is a very useful tool and popular visual plot to display the predicted probabilities of an event for decision support. Therefore, in this study, our aim was to construct and validate a novel nomogram for predicting distant metastasis in T1 and T2 GBC patients using a cohort from the Surveillance, Epidemiology, and End Results (SEER) database.

## Methods

### Patients and Selection Criteria

Gallbladder cancer patient data between 2004 and 2014 were extracted from the SEER registry program of the National Cancer Institute. The SEER database consists of 18 population-based cancer registries among 14 states across the US, including nearly 28% of the US population. The AJCC staging system (6th edition) definitions are as follows: T1 is defined as a tumor invading the lamina propria or muscle layer; T2 is defined as a tumor invading the perimuscular connective tissue, with no extension beyond the serosa or into the liver; N0 means no regional lymph node metastasis; N1 means regional lymph node metastasis; M0 means no distant metastasis; and M1 means distant metastasis.

The inclusion criteria for selection data in this study were as follows: 1) AJCC (6th edition) stage T1 and T2; 2) age >18 years old; 3) diagnosis confirmed by positive histology; and 4) only one primary tumor. The exclusion criteria included the following: 1) data, such as age, sex, T stage and M stage, missing or incomplete; 2) diagnosis before 2004 because the AJCC (6th edition) stage status was not included in the database.

The demographic variables of marital status at diagnosis, age at diagnosis, race, sex, and tumor characteristics of differentiation grade, histological type, T stage, N stage, M stage, and tumor size were collected from the SEER database using SEER-stat software. Because the histological type is mostly adenocarcinoma, the subhistological subtype was classified as adenocarcinoma and others. The SEER database is freely available and has patient anonymization; thus, approval from the ethics committee is not required.

### Construction and validation of the nomogram

All of the eligible T1 and T2 patients extracted from the SEER database were randomly divided into training (60%) and validation (40%) cohorts to establish and validate the nomogram. Furthermore, these two cohorts were divided into two groups according to M stage.

Though univariate analysis, we identified statistically significant variables by screening the clinical characteristics associated with M status. Then, multivariate analysis was used to determine the significant predictive factors among those variables. Finally, on the basis of the analysis results, we used the software R (version 3.6.1) to formulate the nomogram with several packages. To validate the nomogram, a calibration curve with 1000 bootstrap resamples was used in the training set, reflecting the association between the actual probability and predicted probability of positive distant metastasis 8. Moreover, in both the training and validation cohorts, the sensitivity and specificity of the nomogram for predicting distant metastasis were assessed by receiver operating characteristic (ROC) curves and the area under the curve (AUC) value.

### Statistical analysis

SPSS 21.0 statistical software was used for analysis. The method of univariate analysis used was the chi-square test because the data involved in this study are dichotomous variables. Multivariate analysis was carried out by logistic regression, and odds ratios (ORs) and 95% confidence intervals (CIs) were calculated. A *p* value of less than 0.05 was considered significant.

Nomograms from multivariable logistic models are a popular visual plot to display the predicted probabilities of an event for decision support 9. To formulate a nomogram to display the prediction of distant metastasis in early gallbladder carcinoma, the software R 3.6.1 was utilized. The SAS code used in R is according to Lassos A's report 10. Several R packages, including rms, Hmisc, lattice, survival, Formula, and ggplot2, were used to establish the nomogram (available at URL: http://cran.r-project.org/web/packages/).

## Results

### Study cohorts and patient characteristics

**Figure 1:**
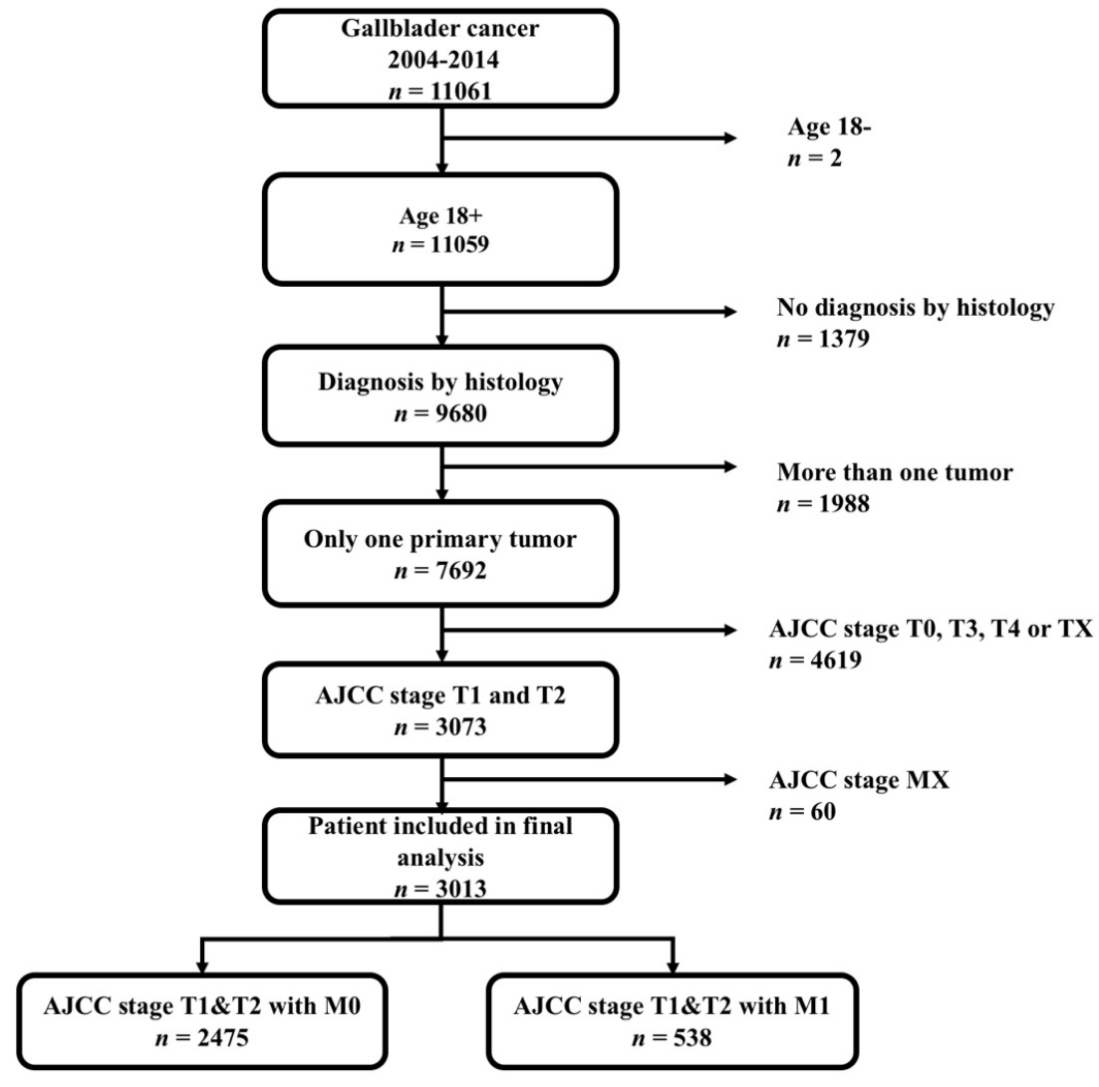
The flow diagram of patient selection.

From 2004 to 2014, 11,061 patients diagnosed with gallbladder cancer were found through the SEER*Stat client-server system. A total of 4619 patients with AJCC stage T0 (*n* =24), T3 (*n* =3154), T4 (*n* =466) and TX (*n* =812, T status data were missing) were excluded; an additional 60 patients with missing M status data were excluded. The flow diagram for the patient selection is presented in Figure 1. Ultimately, 3013 patients with historically confirmed AJCC stage T1 and T2 were identified. Among them, 538 patients (17.9%) were positive for distant metastasis. Eligible patients were randomized and divided into training (*n* = 1808, 60%) and validation (*n*= 1205, 40%) cohorts to formulate and validate the nomogram.

The demographics and clinicopathological characteristics assessed in the three cohorts are summarized in Table 1. The following data were extracted from the SEER database: age, sex, race, marital status, grade, histology, T stage, N stage, M stage and tumor size. In general, the majority of cases were female (2164, 71.8%) and white (2286, 73,7%). Most of the patients had adenocarcinoma (2701, 89.5%). Grade II (1244, 41.2%), T2 (1920, 63.7%), N0 (2261, 75.0%) and larger tumor size (≥ 2, 1517, 50.3%) were more common. The baseline characteristics were balanced between the training and validation cohorts.

### Univariate and multivariate analyses and identification of predictive factors

The results of univariate analysis in the training cohort are listed in Table 2. Age, grade, histological type, T stage, N stage and tumor size were identified as being significantly (*p* < 0.05) associated with M1. All significant factors in the univariate analysis were included in multivariate logistic regression analysis (Table 2), which indicated that younger patients were more likely than older patients to have distant metastasis (age ≤ 70 vs > 70, OR= 0.591, *P*= 0.001). Grade I patients had the lowest risk of M1 (I vs II, III, IV, OR= 2.988, 4.800, 5.573, *P*< 0.001). The risk of M1 in patients with adenocarcinoma was lower than that in patients with other histological types (adenocarcinoma vs others, OR= 1.521, *P*= 0.023). Regarding T and N stage, T1 and N1 were associated with a higher risk of M1 (T1 vs T2, OR= 0.747, *P*= 0.04; N0 vs N1, OR= 3.024, *P*< 0.001). Finally, a larger tumor indicated a higher risk of M1 (<2 vs ≥ 2 cm, OR= 1.578, *P*= 0.004). All the above variables showed a statistically significant predictive capability for M1. After excluding unknown data on grade and tumor size, the remaining factors were selected for building the nomogram.

### Construction and validation of the nomogram

**Figure 2:**
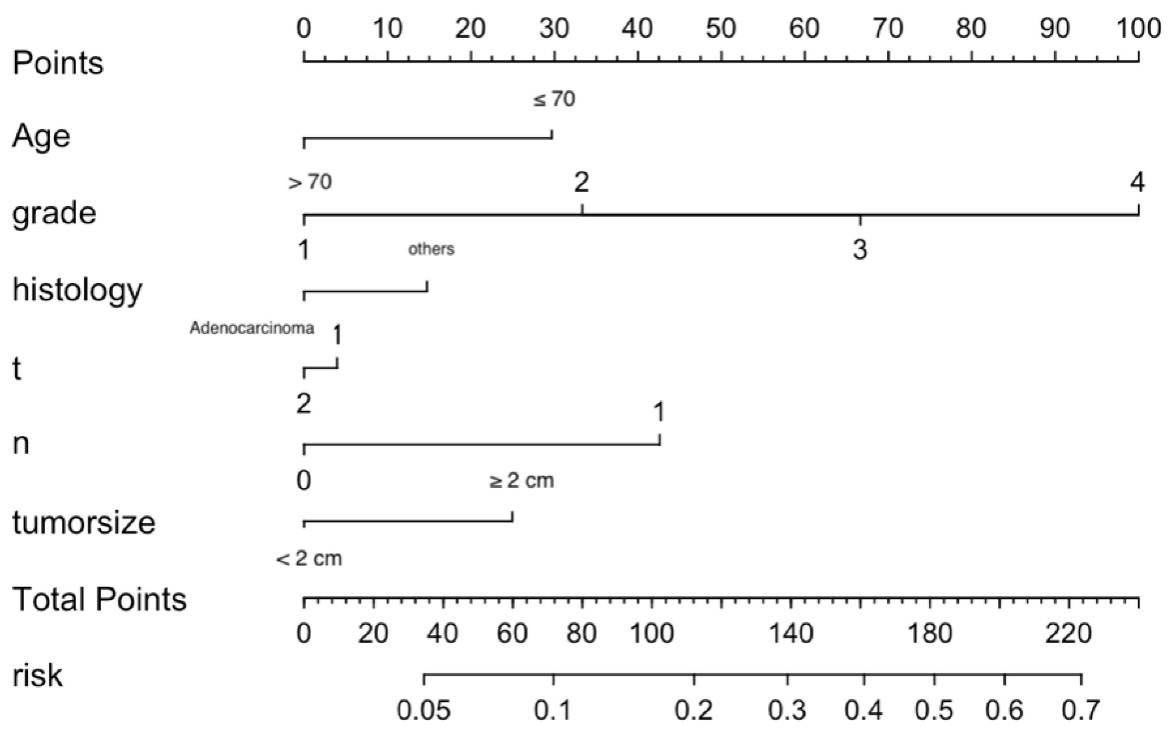
Nomogram predicting the probability of distant metastasis.

Significant independent factors, including age, differentiation grade, histological type, T stage, N stage and tumor size, were incorporated to establish the nomogram (Figure 2). As shown, each factor within these variables was assigned a score on the point of the scale. An age ≤ 70 years old was scored as 30; an age > 70 years old was scored as 0. Differentiation grades I, II, III, and IV were scored as 0, 33.3, 66.7 and 100, respectively. Adenocarcinoma was scored as 0, and others were scored as 15. T1 was scored as 5, and T2 was scored as 0. N1 was scored 45 and N0 0. A tumor size ≥ 2 cm was scored as 25, and a tumor size less than 2 cm was scored as 0. By summing the scores for each variable, we can predict the probability of M1 in a specific patient. For example, a younger patient with nonadenocarcinoma of grade IV, T1, and N1 and larger tumor size (score: 220) had a higher risk of M1. However, a younger adenocarcinoma patient with grade II, T2, and N0 and a smaller tumor (score: 63.3) had a lower risk of M1. The risk of M1 based on our nomogram ranged from 0.05 to 0.7.

**Figure 3:**
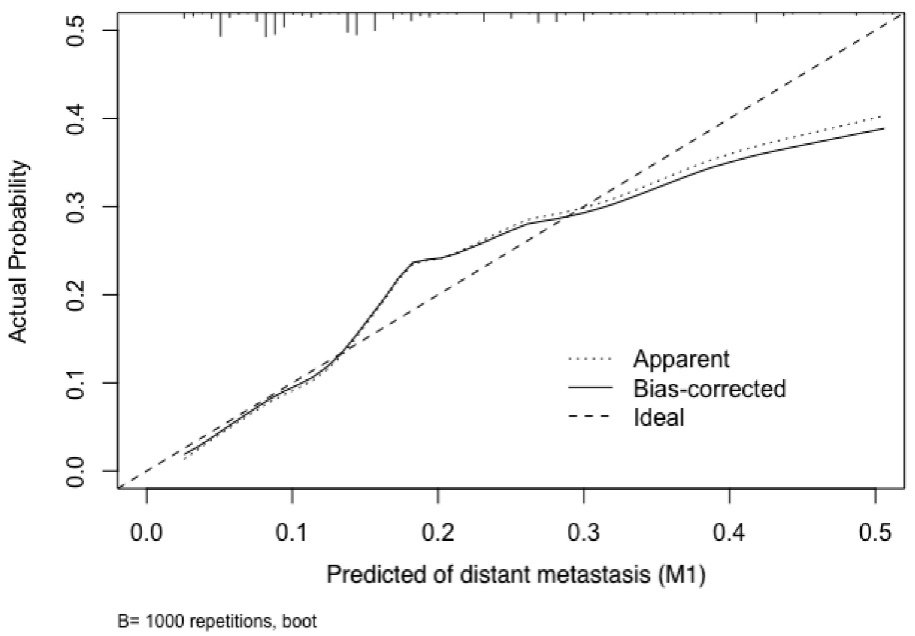
Calibration plot of the nomogram for the probability of distant metastasis (bootstrap 1000 repetitions).

**Figure 4:**
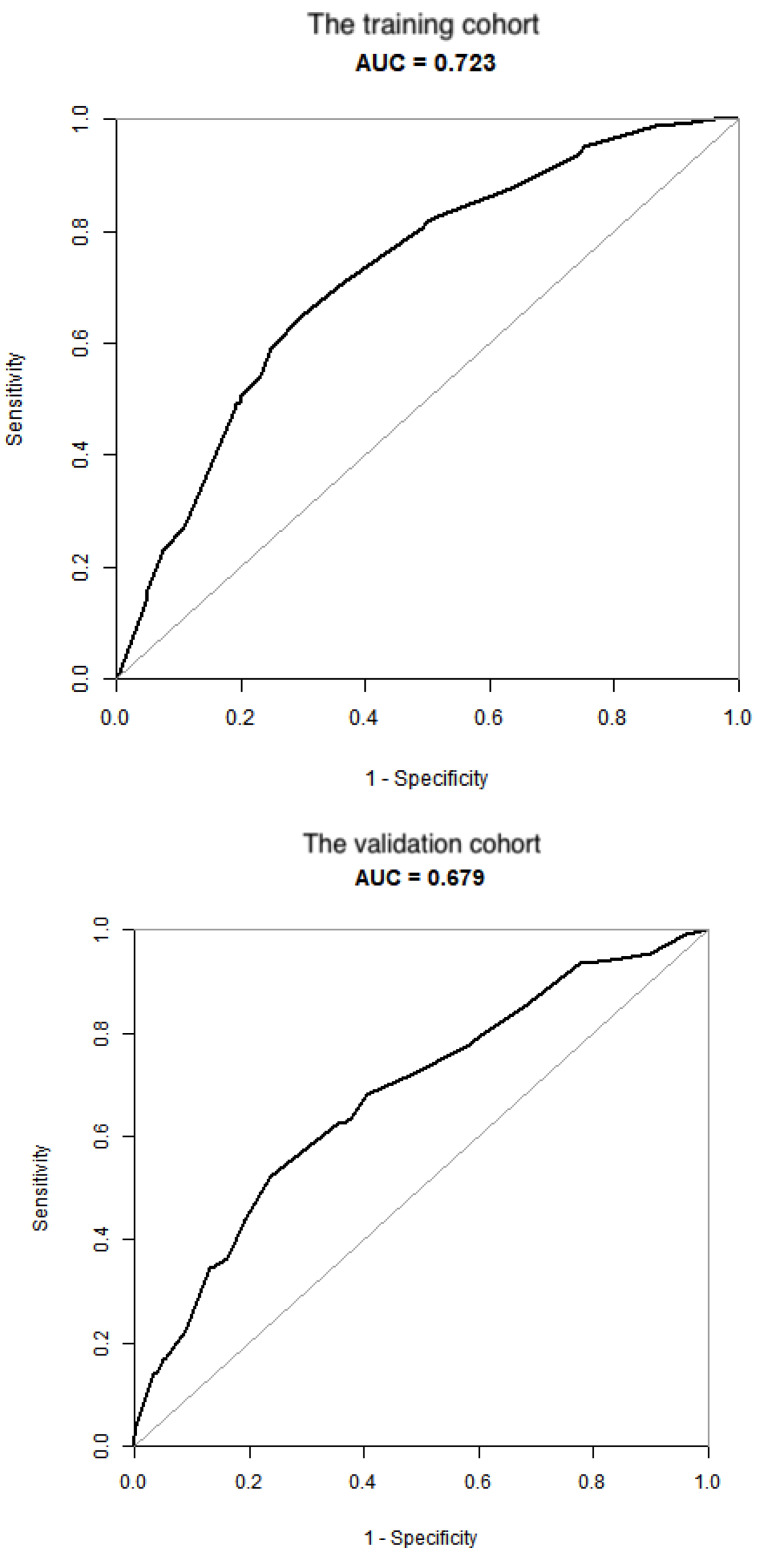
** Validation of the nomogram.** (A) the training cohort; (B) the validation cohort.

To test the performance of the nomogram, it was subjected to 1000 bootstrap resamples for internal validation with a calibration plot in the training cohort (Figure 3). The results showed that this nomogram was well calibrated. Moreover, by using ROC curves, we evaluated the effectiveness of the nomogram in predicting distant metastasis in both the training and validation cohorts (Figure 4A, 4B). In the training cohort, the AUC was 0.723 (95%CI: 0.6865-0.7586, sensitivity: 0.6502, specificity: 0.7009). In the validation cohort, the AUC was 0.679 (95%CI: 0.624-0.7341, sensitivity: 0.5234, specificity: 0.7631). The results further confirmed the effectiveness of the nomogram in predicting M status.

## Discussion

GBC is a dreadful disease because approximately 70% of cases are diagnosed at the regional or distant stage. Most cases at early stages are found incidentally in patients undergoing either laparoscopic or open exploration for cholelithiasis and cholecystitis 11. Although these fortunate patients can be cured by appropriate surgical therapy, some eventually develop distant metastasis. In the present study, 17.9% of early T stage patients (T1 and T2) identified in the SEER database were positive for distant metastasis. This ratio is much higher than expected. Wang *et al* reported that approximately 9.5% of T1b GBC cases were associated with M1, but they only included adenocarcinomas 12. Cecillia G *et al* studied incidental GBC and found distant metastasis in 17% of patients 7. These data, including those from the current study, reveal that one of the biological characteristics of GBC is a tendency toward distant metastasis. Therefore, we developed a predictive nomogram to evaluate the probability of distant metastasis in patients with T1/T2 GBC based on the clinical and pathologic characteristics of the primary tumors.

Based on the statistical analysis, the following six factors were found to be independently associated with M1 risk: age, histological grade, histological subtype, T stage, N stage and tumor size. All these factors were included in this novel nomogram. Regarding the performance of the nomogram, the results of the calibration plot and ROC curves showed satisfactory outcomes. Previous studies have reported that biomarkers with AUCs between 0.7 and 0.9 have superior accuracy, indicating acceptable discrimination 13, 14. In this study, the AUCs in the training and validating cohorts were 0.723 and 0.679, respectively. Therefore, this novel nomogram has moderately accuracy in predicting distant metastasis. Nonetheless, this novel nomogram will help surgeons and oncologists understand the contribution weight of each level of each risk factor for early T stage GBC patients with respect to distant metastasis. Moreover, this model may allow clinicians to screen patients at a higher risk of distant metastasis for closer follow-up and adjuvant treatment.

Regarding the six parameters included in this novel nomogram, histological grade had the highest discriminating power. A poor histological grade usually indicates a higher degree of malignancy, stronger invasive ability, wider range of infiltration, and higher probability of metastasis 15. Our findings support this theory and are consistent with an earlier series from Butte and colleagues, which reported that a poor grade was the strongest predictor of distant metastasis at the time of reresection 16. For differentiation grade, the nomogram demonstrated that N stage was a strong risk factor for M1. Reginal lymph node metastasis (N1) always indicates a worse prognosis among GBC patients 17, 18, and several studies claim that N1 patterns are significant predictors of distant metastasis 19, 20. Our study further confirmed this finding, even among early stages of GBC. In addition, tumor size ≥ 2 cm was significantly associated with an increased risk of distant metastasis. The same pattern was observed in a previous study showing that tumor size > 3 cm indicated worse 1-year and 3-year cancer-specific survival 17. According to this nomogram, patients with age ≤ 70 had a higher probability of distant metastasis. Similar results have been found in breast cancer patients. E Colzani *et al* suggested that breast cancer patients younger than 50 years at diagnosis had a higher risk of distant metastasis 21. However, among patients with squamous cell cancer of the head and neck, Kuperman *et al* reported that advanced age was associated with an increased risk of distant metastasis^22^.The inconsistent results in head and neck tumors indicate that younger patients have a higher risk of distant metastasis is a characteristic of GBC and breast cancer. In addition, the study of Somasundar *et al* reported that young age (under 50 years) is a poor prognostic factor in gallbladder cancer 23. This research supports that our results are convincing and shows one characteristic of gallbladder cancer. With regard to histological type, our study found that nonadenocarcinoma was associated with an increased likelihood of distant metastasis. Nonadenocarcinoma is rare among GBC patients, and most of the studies to date are case reports. Samuel *et al* performed a study on the clinicopathological characteristics and outcomes of rare histologic variants of gallbladder cancer and reported that adenosquamous/squamous GBC had worse survival outcomes than gallbladder adenocarcinoma 24. Furthermore, the analysis of 34 adenosquamous/squamous GBC cases and comparison of clinicopathologic features and surgical outcomes with adenocarcinoma by Wang *et al* showed that adenosquamous/squamous GBC tended to be associated with more infiltration of multiple adjacent organs and lymphatic metastasis 25. Our research is consistent with the above studies. Finally, an interesting finding was that stage T1 patients had a higher risk of M1 than stage T2 patients. This was inconsistent with previous research 7, 16. In our view, this is because we excluded patients without a histological diagnosis. T1 patients can undergo surgery, and distant metastasis is incidentally found; T2 patients with distant metastasis might not have the chance to receive surgery and obtain a histological diagnosis, and they would have been excluded from this study. Furthermore, we performed subgroup analysis between T1 and T2 patients without histologic confirmation and found no significant difference in distant metastasis, supporting our explanation. Moreover, T stage had the lowest discriminating power in this nomogram, with little impact on the final nomogram results.

With regard to the clinical utility of our nomogram, the unique visual effect of this predictive model will help surgeons intuitively understand the risk level of the above factors regarding distant metastasis. After the diagnosis of GBC has been confirmed by pathologic examination, guidelines highly recommend that appropriate staging be performed prior to initiating treatment 26. PET scans may alter the management of 23% of patients with GBC 27, and 20% of patients who underwent staging laparoscopy prior to laparotomy had distant disease 16. Thus, PET-CT or staging laparoscopy may be considered for patients at higher risk of M1 based on our nomogram. On the other hand, disease recurrences, such as distant metastasis, often occur after resection, which highlights the systemic nature of GBC and the need for multimodality therapy 28. Although an ideal regimen of adjuvant therapy has not been developed, recent clinical trials and studies have shown that the use of postoperative chemoradiotherapy can improve oncologic outcomes. Indeed, one population-based study that analyzed 18,124 cases from 1973 to 2009 concluded that the highest survival was associated with receiving both surgery and radiation 2. The BILCAP (Biliary Capecitabine) randomized controlled trial involving 447 patients with resected biliary tract malignancies reported that 6 months of adjuvant capecitabine improved overall survival compared to placebo 29. A phase II trial reported a well-tolerated adjuvant regimen of gemcitabine and capecitabine with radiotherapy and showed a 2-year overall survival of 67% and a median overall survival of 35 months 30. Through this predictive model, surgeons and oncologists will be able to estimate the possibility of distant metastasis in early T stage GBC patients. Thus, we believe that postoperative chemoradiotherapy should be considered for patients with a higher possibility of distant metastasis, or at least a much closer follow-up should be considered.

Several limitations exist in our study. First, the SEER database did not include records of lymphovascular (LVI) and perineural (PNI) invasion. As LVI and PNI are associated with increased rates of distant metastasis 7, including these two predictive factors may improve the sensitivity and specificity of our nomogram. Second, this study was a retrospective study, which is associated with inevitable selection bias and information bias. Third, we evaluated GBC patients between 2004 and 2014 to develop the nomogram; thus, the staging system used in this nomogram is the AJCC 6th edition provided by the SEER database (N stage only included N0 and N1). The latest edition is 8th, which has changed the definition of N stage based on the number of regional lymph node metastases. This difference may influence the accuracy of the nomogram. Fourth, the AUC from the validation cohort was below 0.7 (0.679), which revealed that the novel nomogram only has moderately accuracy in predicting distant metastasis. Because the accuracy was not strong enough, doctors should pay attention to its limitations when using the nomogram. Finally, although this novel predictive model was established by a large cohort and validated in a split subgroup of patients, external validation of the nomogram is still necessary.

## Conclusion

In conclusion, through retrospective analysis of 3013 patients with T1/T2 GBC, this study established a novel nomogram based on six independent risk factors to predict distant metastasis. The validation of the model proved its satisfactory performance. Although some limitations exist in this predictive model, the nomogram revealed the relationship between the clinicopathological characteristics of T1 and T2 GBC patients and the risk of distant metastasis. This tool will assist in patient counseling and guide treatment decision making for T1 and T2 GBC patients. In the future, more randomized controlled trials are needed to provide accurate data to improve and update this nomogram.

## Figures and Tables

**Table 1:** Demographics and clinical characteristics of the whole, training and validation cohorts.

Variables	Whole cohort (*n* = 3013)		Training cohort (*n* = 1808)		Validation cohort (*n* = 1205)	
	M0 (*n* = 2475)	M1 (*n* = 538)	M0 (*n* = 1458)	M1 (*n* = 350)	M0 (*n* = 1017)	M1 (*n* = 188)
**Age (years)**						
≤ 70	1185 (47.8%)	300 (55.7%)	709 (48.6%)	202 (57.7%)	476 (46.8%)	98 (52.1%)
> 70	1290 (52.2%)	238 (44.3%)	749 (51.4%)	148 (42.3%)	541 (53.2%)	90 (48.9%)
**Sex**						
Male	699 (28.2%)	150 (27.9%)	402 (27.6%)	91 (26.0%)	297 (29.2%)	59 (31.4%)
Female	1776 (71.8%)	388 (72.1%)	1056 (72.4%)	259 (74.0%)	720 (70.8%)	129 (68.6%)
**Race**						
White	1883 (76.1%)	403 (74.9%)	1126 (77.3%)	254 (72.6%)	757 (74.4%)	149 (79.3%)
Black	293 (11.8%)	73 (13.6%)	161 (11.0%)	53 (15.1%)	132 (13.0%)	20 (10.6%)
Others^a^	299 (12.1%)	62 (11.5%)	171 (11.7%)	43 (12.3%)	128 (12.6%)	19 (10.1%)
**Marital status**						
Married	1217 (49.1%)	280 (52.0%)	725 (49.7%)	167 (47.7%)	492 (48.3%)	113 (60.1%)
Unmarried^b^	1258 (50.9%)	258 (48.0%)	733 (50.3%)	183 (52.3%)	525 (51.7%)	75 (39.9%)
**Grade**						
I	516 (20.8%)	31 (5.8%)	279 (19.1%)	14 (4.0%)	237 (23.3%)	17 (9.0%)
II	1074 (43.4%)	170 (31.6%)	632 (43.3%)	108 (30.8%)	442 (43.5%)	62 (33.0%)
III	626 (25.3%)	186 (34.6%)	391 (26.9%)	127 (36.3%)	235 (23.1%)	59 (31.4%)
IV	34 (1.4%)	15 (2.8%)	20 (1.4%)	8 (2.3%)	14 (1.4%)	7 (3.7%)
Unknow	225 (9.1%)	136 (25.2%)	136 (9.3%)	93 (26.6%)	89 (8.7%)	43 (22.9%)
**Histology**						
Adenocarcinoma	2264 (91.5%)	437 (81.2%)	1330 (91.2%)	285 (81.4%)	934 (91.8%)	152 (80.9%)
Others^c^	211 (8.5%)	101 (18.8%)	128 (8.8%)	65 (18.6%)	83 (8.2%)	36 (19.1%)
**T stage**						
T1	863 (34.9%)	230 (42.8%)	510 (35.0%)	153 (43.7%)	353 (34.7%)	77 (40.9%)
T2	1612 (65.1%)	308 (57.2%)	948 (65.0%)	197 (56.3%)	664 (65.3%)	111 (59.1%)
**N stage**						
N0	1957 (79.0%)	304 (56.5%)	1149 (78.8%)	183 (52.3%)	808 (79.4%)	121 (64.4%)
N1	518 (11.0%)	234 (43.5%)	309 (11.2%)	167 (47.7%)	209 (10.6%)	67 (35.6%)
**Tumor size**						
< 2	800 (32.3%)	116 (21.6%)	484 (33.2%)	78 (22.3%)	316 (31.1%)	38 (20.2%)
≥ 2	1217 (49.2%)	300 (55.8%)	706 (48.4%)	200 (57.1%)	511 (50.2%)	100 (53.2%)
Unknow	458 (18.5%)	122 (22.6%)	268 (18.4%)	72 (20.6%)	190 (18.7%)	50 (26.6%)

Data expressed as a number (%). ^a^Includes: American Indian/native Alaskan and Asian/Pacific Islander; ^b^Includes: divorced, separated, single, domestic partner and widowed; ^c^Includes: epithelial neoplasms, cystic, mucinous and serous neoplasms, squamous cell neoplasms.

**Table 2:** Univariate and multivariate analysis in the training cohort.

Variables	Univariate analysis *p* value		Multivariate analysis		Selected factors for building nomogram		
	*P*	OR	95% CI	*P*	OR	95% CI	*P*
**Age (years)**	0.002						
≤ 70		1 (reference)			1 (reference)		
> 70		0.591	0.433-0.805	0.001	0.591	0.433-0.805	0.001
**Sex**	0.553						
Male							
Female							
**Race**	0.087						
White							
Black							
Others^a^							
**Marital status**	0.499						
Married							
Unmarried^b^							
**Grade**	< 0.001						
I		1 (reference)					
II		2.988	1.669-5.348	< 0.001	2.988	1.669-5.348	< 0.001
III		4.800	2.655-8.646	< 0.001	4.800	2.655-8.646	< 0.001
IV		5.573	2.004-15.498	0.001	5.573	2.004-15.498	0.001
Unknow		9.562	136 (59.4%)	< 0.001			
**Histology**	< 0.001						
Adenocarcinoma		1 (reference)					
Others^c^		1.521	1.061-2.180	0.023	1.521	1.061-2.180	0.023
**T stage**	0.002						
T1		1 (reference)					
T2		0.747	0.565-0.987	0.04	0.747	0.565-0.987	0.04
**N stage**	< 0.001						
N0		1 (reference)					
N1		3.024	2.323-3.938	< 0.001	3.024	2.323-3.938	< 0.001
**Tumor size**	< 0.001						
< 2		1 (reference)					
≥ 2		1.578	1.160-2.148	0.004	1.578	1.160-2.148	0.004
Unknow		2.232	1.428-3.488	< 0.001			

Data expressed as a number (%). ^a^Includes: American Indian/native Alaskan and Asian/Pacific Islander; ^b^Includes: divorced, separated, single, domestic partner and widowed; ^c^Includes: epithelial neoplasms, cystic, mucinous and serous neoplasms, squamous cell neoplasms.

## Abbreviations

GBC: Gallbladder cancer
SEER: Surveillance, Epidemiology, and End Results database
ROC: receptor operating characteristic curve
